# From incidental findings to diagnosis: Adult presentation of asymptomatic congenital lobar emphysema

**DOI:** 10.1016/j.rmcr.2025.102231

**Published:** 2025-05-14

**Authors:** Reza jalli, Mohammad Javad Fallahi, Seyed Sina Dehghani

**Affiliations:** a Medical Imaging Research Center, Shiraz University of Medical Sciences, Shiraz, Iran; b Pulmonology Division, Department of Internal Medicine, Shiraz University of Medical Sciences, Shiraz, Iran; c Department of Radiology, Shiraz University of Medical Sciences, Shiraz, Iran

**Keywords:** Congenital lobar emphysema, Adult-onset pulmonary anomalies, Incidental imaging findings

## Abstract

Congenital lobar emphysema (CLE) is a rare congenital lung anomaly that is typically diagnosed in infancy. Adult presentations are exceptionally uncommon, and most cases are discovered incidentally during imaging for unrelated conditions. We present two cases of CLE diagnosed in asymptomatic adults, emphasizing the critical role of imaging in diagnosis and outlining the considerations for conservative management. These cases contribute to the understanding of CLE in adult patients.

## 1 Introduction

**Fig. 1:**
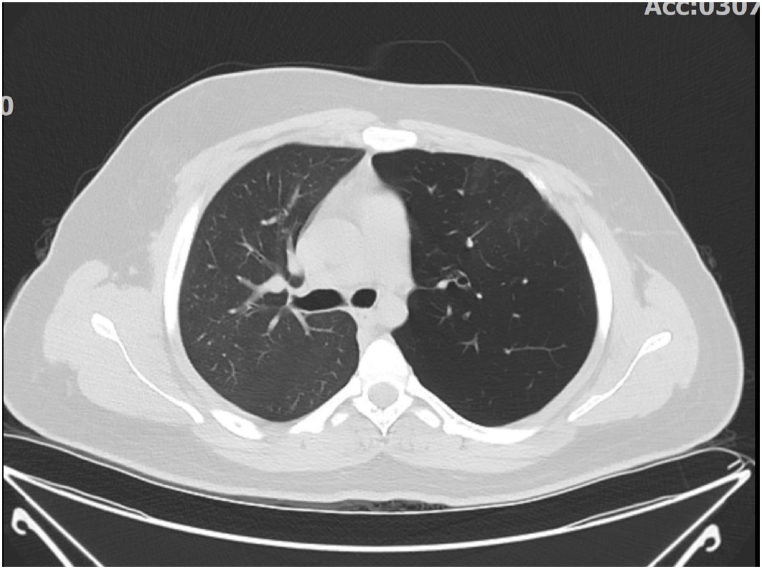
Axial HRCT. Axial view highlights expansion of the left upper lobe with rightward mediastinal displacement.

**Fig. 2:**
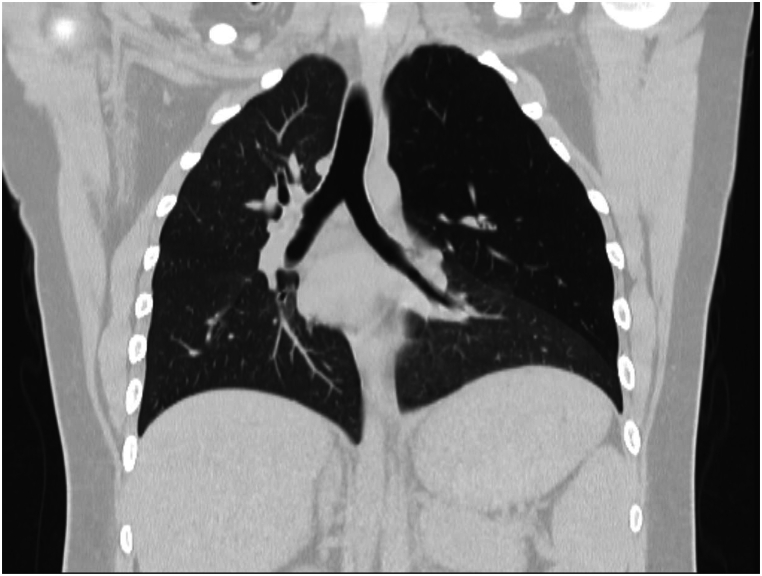
Coronal HRCT. Entire part of left upper lobe including superior and inferior segments of lingula demonstrates hyperinflation associated with diminished lung markings. Pressure effect over mediastinal structures is seen secondary to ball-valve mechanism.

**Fig. 3:**
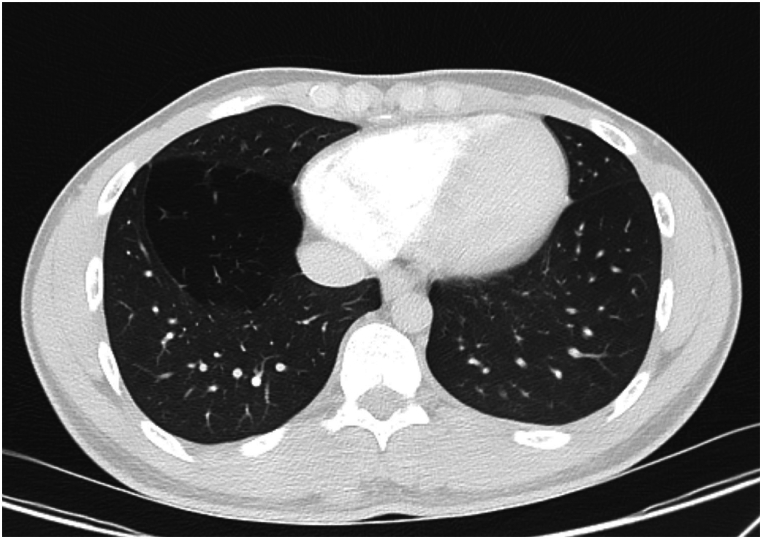
Axial CT image. Hyperinflated medial basal segment of right lower lobe.

Congenital lobar emphysema (CLE), sometimes referred to as congenital lobar overinflation, is a rare condition marked by abnormal hyperinflation of one or more lung lobes, typically caused by partial bronchial obstruction. While primarily diagnosed in infancy, typically With in the first year of life due to severe respiratory distress, CLE may remain asymptomatic or undiagnosed until adulthood, especially in cases with milder presentations or incidental imaging findings [1,2]. Adult presentations are exceptionally rare, making CLE a diagnostic challenge for clinicians and radiologists alike [3] (see Fig. 1, Fig. 2, Fig. 3).

Given its rarity in adults, CLE poses unique diagnostic and management challenges. This case report aims to highlight two adult cases of CLE, emphasizing the clinical presentation, imaging findings, and treatment strategies, contributing to the limited literature on this topic.

## 2 Case presentation

We report two cases of incidental CLE diagnosed in asymptomatic young man patients during imaging for unrelated conditions.

### 2.1 Case 1

A 19-year-old male, nonsmoker with no significant medical history presented with complaints of upper abdominal pain. An abdominal CT scan revealed a suspicious finding in the lung base, prompting further evaluation with a high-resolution computed tomography (HRCT) of the chest.

HRCT demonstrated hyperinflation and hyperlucency of the left upper lobe (LUL), with resultant mediastinal shift to the right and compression of the left lower lobe. There were no signs of bullous lesions, masses, or pleural effusion. The patient had no respiratory symptoms (e.g., dyspnea, cough) and was managed conservatively with regular follow-up.

To rule out intrinsic causes of airway abnormalities as well as intraluminal lesions, fiberoptic bronchoscopy (FOB) was performed. No abnormalities were detected in the lobar and segmental branches of the tracheobronchial tree.

### 2.2 Case 2

A 19-year-old male, nonsmoker presented for preoperative evaluation prior to planned inguinal hernia repair. During the surgical workup, a chest CT scan was performed, which revealed incidental findings in the right lung. The CT scan demonstrated hyperlucency and overinflation of the right lower lobe (RLL). There was evidence of.

1. Compression of adjacent lung parenchyma, particularly the right lower lobe.
2. Absence of bullous lesions, masses, or pleural effusion.

The patient remained asymptomatic, reporting no history of respiratory distress, chronic cough, or recurrent infections. Given the incidental nature of the diagnosis and the absence of symptoms, conservative management with regular imaging follow-up was recommended. In this patient, a segment of the lower lobe is involved, but no lesion was observed in the airway of this segment during fiberoptic bronchoscopy (FOB).

## 3 Discussion

Congenital lobar emphysema (CLE) is a rare developmental anomaly of the lung characterized by hyperinflation of one or more pulmonary lobes due to partial obstruction or malformation of the bronchial cartilage [4,5]. It is typically diagnosed in infancy or early childhood when symptoms such as respiratory distress, tachypnea, or failure to thrive become apparent. However, CLE can occasionally remain asymptomatic and may be discovered incidentally in adulthood, as seen in our case.

The underlying etiology of CLE involves a multifactorial process, most commonly attributed to defects in bronchial cartilage that result in expiratory airway collapse and subsequent air trapping. Additional causes include external bronchial compression by vascular structures or abnormal bronchial wall dynamics [6,7]. The left upper lobe is most frequently affected (43 %), followed by the right middle lobe (32 %), right upper lobe (21 %); however, the lower lobes involvement is uncommon [8].

From a radiological perspective, CLE presents as pronounced overinflation of the affected lung lobe, often causing compression of nearby lung tissue and a mediastinal shift. Chest radiographs often reveal hyperlucency of the affected lobe, while CT imaging is indispensable for confirming the diagnosis, ruling out mimickers such as bullous emphysema, and assessing complications [7,8].

CLE presenting in adulthood is exceptionally rare and poses a diagnostic challenge. While infants typically present with respiratory distress, adults are often asymptomatic or present with nonspecific symptoms such as chronic cough, dyspnea, or recurrent respiratory infections [9]. In our case, the diagnosis was incidental following evaluation for other diseases, which highlights the importance of carefully reviewing all lung fields during routine imaging.

CT scan plays a critical role in diagnosing CLE, particularly in adults where other causes of lobar hyperinflation must be excluded. Imaging findings include.

• Marked hyperlucency of the affected segment.
• Expansion of the lobe, with compression over adjacent lung parenchyma.
• Mediastinal shift away from the affected lobe due to volume expansion [10].

In CLE, lack of other features such as bullous lesions, pulmonary masses, or pleural effusion helps differentiate it from other conditions like bullous emphysema, pneumothorax, or large cystic lesions [11].

The differential diagnosis for lobar hyperinflation includes.

1. Bullous emphysema: Usually associated with chronic smoking and presents as localized areas of destruction with thin walls.
2. Pneumothorax: Demonstrates air in the pleural cavity and a clear pleural line.
3. Bronchial obstruction (e.g., foreign body, tumor): May cause distal hyperinflation but usually involves atelectasis or air trapping.
4. Large cystic lung disease: Includes congenital pulmonary airway malformation (CPAM) and pulmonary sequestration [12].

Management of CLE depends on the severity of symptoms and the degree of lung involvement.

• In symptomatic cases with respiratory compromise, lobectomy is often performed to relieve symptoms and prevent complications such as recurrent infections or pneumothorax [13].
• In asymptomatic patients, such as our cases, conservative management with regular clinical and radiological follow-up is sufficient [14].

The incidental diagnosis of CLE in asymptomatic adults, while rare, demonstrates the benign nature of the condition in some cases and reinforces the importance of accurate imaging interpretation.

## 4 Conclusion

These two cases demonstrate the importance of recognizing congenital lobar emphysema as an incidental finding in adults, particularly during imaging for unrelated conditions. Despite its rarity, CLE should remain in the differential diagnosis for lobar or segmental hyperinflation of the lungs. Accurate imaging interpretation is critical to avoid unnecessary interventions and guide appropriate management.

## CRediT authorship contribution statement

**Reza jalli:** Supervision, Validation. **Mohammad Javad Fallahi:** Conceptualization, Resources, Supervision. **Seyed Sina Dehghani:** Writing – original draft, Writing – review & editing.

## Ethical considerations

Written informed consent was obtained from both patients for the publication of this case report, including the use of accompanying images. This report complies with the ethical standards set forth in the Declaration of Helsinki.

## Declaration of competing interest

The authors declare that they have no known competing financial interests or personal relationships that could have appeared to influence the work reported in this paper.
